# Social Media Disorder, Mental Health, and Validation of the Chinese Version of 27-Item Social Media Disorder Scale in Chinese College Students

**DOI:** 10.3389/fpubh.2022.942720

**Published:** 2022-07-05

**Authors:** Hui Lei, Yaqing Huang, Ya Chai, Xiaocui Zhang

**Affiliations:** ^1^College of Education, Hunan Agricultural University, Changsha, China; ^2^Center for Magnetic Resonance Imaging Research and Key Laboratory of Applied Brain and Cognitive Sciences, School of Business and Management, Shanghai International Studies University, Shanghai, China; ^3^Medical Psychological Center, The Second Xiangya Hospital, Central South University, Changsha, China; ^4^Medical Psychological Institute of Central South University, Changsha, China; ^5^National Clinical Research Center for Mental Disorders, Changsha, China

**Keywords:** social media addiction, social media disorder, mental health, college students, psychometric validation

## Abstract

**Objective:**

With the widespread use of social media, excessive use of social media may lead to problematic behaviors such as social media disorder, which has a negative impact on teenagers' mental health. Thus, it is an urgent need to provide a measurement tool to assess social media addiction in different cultures. The aim of this study was to assess the psychometric properties of the Chinese version of 27-item Social Media Disorder (SMD) Scale (developed using the diagnostic criteria of DSM-V Internet Gaming Disorder) in college students, and to verify its impact on mental health.

**Methods:**

Two online surveys were conducted among a total of 1,539 Chinese college students, including 1,316 subjects in sample 1 and 223 subjects in sample 2. The discrimination, criterion validity, construct validity and reliability of the Chinese version of SMD-27 scale were examined.

**Results:**

The Chinese version of SMD-27 scale showed excellent psychometric properties. The item-total correlation coefficients of the scale ranged from 0.31 to 0.56, and the item-dimension correlations of the scale ranged from 0.459 to 0.834. Findings from confirmatory factor analysis indicated a great fit of the model of the Chinese version of SMD-27, with *CFI* = 0.956, *TLI* = 0.951, *RMSEA* = 0.036 in sample one and *CFI* = 0.970, *TLI* = 0.967, *RMSEA* = 0.040 in sample two, thus confirming the second-order factor structure of the scale. The SMD-27 scale showed good internal consistency between two different samples with their respective Cronbach's alpha of 0.87 and 0.92, and good test-retest reliability over a period of 1 month. In addition, multiple regression results generally supported the impact of social media addiction on mental health.

**Conclusion:**

This study provides evidence that the Chinese version of SMD-27 scale is applicable to Chinese college populations, and it is a promising tool for the study of social media addiction in China.

## Introduction

With the rapid development of Internet technology and the popularity of smart phones, social media gradually plays an important role in people's interpersonal communications. In 2020, over 3.6 billion people were using social media worldwide, a number projected to increase to almost 4.41 billion in 2025 (1). Using social media platforms is more common among adolescents, 71% of whom report using more than one social media platform (2). However, excessive use of social media may lead to problematic behaviors such as social media disorder (SMD), which may have a negative impact on adolescents (3).

SMD is a behavioral addition, which refers to an individual's excessive attention and investment of time and energy in social media, driven by a strong motivation to use or log on to social media (4). Though has not been defined as a disorder in DSM-5, SMD has symptoms similar to other addictive behaviors (5), such as mood modification, salience tolerance, withdrawal and emotional symptoms (6).

China is the world's largest social media market with highly engaged and mobile-savvy user (1). College students have a high incidence of SMD (7). Studies have shown that the rate of social network addiction among Chinese college students is as high as 20.9% (8), which seriously affects their academic performance (9). Increasing evidence has suggested that individuals with SMD tend to have low emotional regulation ability (10), and excessive social media use brought about emotional exhaustion and fatigue (11), as well as increased risk of depression and anxiety (12–14). Thus, it is an urgent need for researchers and medical practitioners to develop a well-defined, reliable, and validated instrument to measure SMD.

Various psychometric instruments that measure social media disorder have been developed, such as the Bergen Social Media Addiction Scale (15), Facebook Intrusion Questionnaire (16), and Social Networking Website Addition Scale (17). However, these scales focused on distinguishing between disordered and highly engaged non-disordered social media users. A promising scale, the SMD scale, was recently developed to measure social media disorder. The SMD scale was based on the DSM-5 criteria, and included nine indicators, which were preoccupation, tolerance, withdrawal, persistence, escape, problems, deception, displacement and conflict. Moreover, it was found to be more consistent with the clinical definition of behavioral addiction (18). Hence, the SMD scale appears to be more comprehensive than the existing scales. The 9-item SMD scale has been validated in adolescent groups in China (19), Turkish (20), and Netherlands (21), as well as across 44 countries (22), suggesting the validity of the SMD-9 scale in measuring problematic usage of social media in adolescents. Furthermore, the 27-item SMD scale, consist of three items for each of the nine DSM-5 criteria, has been found to be more specific and comprehensive in describing social media addiction behavior as compared to SMD-9 scale. Therefore, the validity of the SMD-27 scale is worth studying in Chinese culture, though its validity has been confirmed among over 2000 Dutch adolescents (18).

The main purpose of this study is to evaluate the psychometric properties of the Chinese version of SMD-27 scale and the impact of social media addiction on the mental health of college students. It is hoped to provide a reliable and effective tool for the measurement of social media addiction in China and promote the related research on social media addiction in China.

## Methods

### Sample and Procedure

Two online surveys were conducted among a total of 1,539 Chinese college students who were recruited from five colleges and universities in Hunan province and Guangxi Province. The first survey was distributed among 1,316 participants. One thousand two hundred and ninety-eight valid questionnaires were collected that the effective rate was 98.63%. Sample 1 included 346 boys (26.7%) and 952 girls (73.3%). There were 635 freshmen (48.8%), 287 sophomores (22.1%), 327 juniors (25.2%), and 49 seniors (3.8%). The mean age of the sample was 19.11 ± 1.24 years, with the range from 16 to 24.

The second survey was conducted among a new sample of 223 subjects. Sample 2 included 192 girls (86.1%) and 31 boys (13.9%), and the mean age of the sample was 19.75 ± 1.21 years. One month later, 169 students completed the follow-up survey, including 149 girls (88.2%) and 20 boys (11.8%) with an average age of 19.67 ± 1.16 years, with the range from 17 to 23.

Before administration, informed consent was obtained from all subjects. The English SMD-27 scale was translated into Chinese in a three-step process after obtaining the authorization of the original author. Firstly, the English SMD-27 scale was translated into two Chinese versions independently by two bilingual graduate students majoring in psychology. Secondly, a synthesized Chinese version was generated by the two translators and a psychologist. Finally, the synthesized Chinese version was translated back into English by one English native translator who was fluent in Chinese. Then an expert committee including two psychologists and a psychiatrist reviewed all translated versions and finalized the Chinese version based on the cultural background and language habits in China.

### Measures

#### The 27-Item Social Media Disorder Scale

The 27-item SMD scale consists of nine dimensions, with three items representing one dimension. These dimensions include preoccupation, tolerance, withdrawal, persistence, escape, problems, deception, displacement, and conflict. For analysis, the answers of “yes” were summed up, resulting in a total score of the SMD-27 scale ranging from 0 to 27 (yes = 1, no = 0) with higher scores indicating more severe social media addition (18).

#### Problematic Mobile Social Media Usage Assessment Questionnaire for Adolescents (PMSMU)

The PMSMU consists of 20 items that are answered on a five-point Likert scale from strongly disagree to strongly agree (23). It includes five factors: viscosity increase, physiological damage, omission anxiety, cognitive failure, and guilt. The questionnaire has good structural validity. Higher scores indicate a greater severity of problematic mobile social media usage. The Cronbach's alpha for this scale was 0.94 in sample 1.

#### Patient Health Questionnaire-9 (PHQ-9)

The PHQ-9 was used to measure depression, with response categories ranging from 0 (never) to 3 (very often). Item scores of the scale were added up to generate the total score. Higher scores indicate a greater severity of depression (24). The Cronbach's alpha for this scale was 0.93 in sample 1.

#### Interaction Anxiousness Scale (IAS)

The IAS consists of 15 items that are answered on a five-point Likert scale from strongly disagree to strongly agree. Higher score indicates higher level of anxiety (25). The Cronbach's alpha for this scale was 0.84 in sample 1.

#### Fatigue Severity Scale (FSS)

The FFS consists of nine items that are answered on a seven-point Likert scale from totally disagree to totally agree. There are three grades: no fatigue (<4 points), moderate fatigue (4–4.9 points), and severe fatigue (≥5 points) (26). The Cronbach's alpha for this scale was 0.89 in sample 1.

### Statistical Analysis

The item analysis, reliability analysis, convergent and criterion validity of the SMD-27 scale was performed with SPSS Statistics 25.0. Confirmatory factor analysis was performed with Mplus7.0.

In order to verify the discrimination of the SMD-27 scale, we divided the participants into a “low” group and a “high” group based on the total score and used the ratio 27% to determine the lower and upper groups, and then performed independent samples *T*-test on these two groups. Then we conducted Pearson correlation analysis between the score of each item and the total score of the scale. The internal consistency reliability of the scale was assessed using Cronbach's alpha coefficient, and values >0.7 were considered acceptable.

To assess the construct validity of the SMD-27 scale, confirmatory factor analysis (*CFA*) was performed using the robust weighted least squares estimator. χ^2^ and its degrees of freedom (*df*) were used to evaluate the good-fitting of the model with χ^2^/*df* ≤ 3 indicating an acceptable model given the large sample size (27). In addition, the comparative fit index (*CFI*), Tucker Lewis index (*TLI*), and root mean square error of approximation (*RMSEA*) with confidence intervals (*CI*) were also used (*CFI/TLI* ≥ 0.90 acceptable, ≥0.95 good; *RMSEA* ≤ 0.08 acceptable, ≤ 0.06 good) (28). Criterion validity was assessed with other addiction-related scales.

Pearson correlation analyses were performed to examine the associations between SMD, depression, anxiety, and fatigue. To examine the impact of social network addiction on mental health, we included the nine dimensions of SMD-27 scale, age, and gender as independent variables, and three mental health scales (i.e., depression, anxiety, and fatigue) as three dependent variables, to perform multiple regression analyses using the stepwise method.

## Results

### Social Media Use

The results on social media use was derived from sample 1 (*N* = 1,298). All participants reported using at least one form of social media. Among all social media users, 99.61% reported using smartphones. The most popular social media platforms were Wechat (95.1%) and QQ (91.1%), followed by Weibo (49.8%), Tiktok (44.2%), and Kuaishou (14.3%).

### Item Analysis

To examine the item discrimination of the SMD-27 scale in sample 1 (*N* = 1,298), the total scores of the SMD-27 scale were arranged in descending order, with the top 27% as high group and the bottom 27% as low group. Results from independent samples *T*-test showed that there were statistically significant differences between the two groups (*t* = 9.34–28.00, *p* < 0.001). The percentage of affirmative answers in all 27 items ranged from 6.2 to 61.6%. In addition, the item-total correlation coefficients of the scale ranged from 0.31 to 0.56 (*p* < 0.001), and the item-dimension correlation coefficients of the scale ranged from 0.459 to 0.834, which are within the acceptable level (Table 1). These results indicated that the items have a good discrimination.

**Table 1:** Descriptive statistics for the SMD-27 scale (*N* = 1,298).

**Item**	***Mean***	***SD***	**% yes**	***t***	***r_**1**_***	***r_**2**_***
Preoccupation1	0.567	0.496	56.7	18.340^***^	0.454^***^	0.719^***^
Preoccupation2	0.204	0.403	20.4	16.564^***^	0.493^***^	0.702^***^
Preoccupation3	0.216	0.411	21.6	16.543^***^	0.469^***^	0.676^***^
Tolerance1	0.431	0.495	43.1	24.106^***^	0.534^***^	0.779^***^
Tolerance2	0.594	0.491	59.4	23.458^***^	0.503^***^	0.776^***^
Tolerance3	0.208	0.406	20.8	18.471^***^	0.548^***^	0.678^***^
Withdrawal1	0.222	0.416	22.2	17.429^***^	0.485^***^	0.767^***^
Withdrawal2	0.126	0.332	12.6	13.484^***^	0.491^***^	0.743^***^
Withdrawal3	0.292	0.455	29.2	20.046^***^	0.515^***^	0.824^***^
Persistence1	0.376	0.485	37.6	22.678^***^	0.529^***^	0.834^***^
Persistence2	0.381	0.486	38.1	25.360^***^	0.550^***^	0.834^***^
Persistence3	0.287	0.452	28.7	23.624^***^	0.564^***^	0.766^***^
Escape1	0.473	0.499	47.3	28.000^***^	0.560^***^	0.766^***^
Escape2	0.616	0.486	61.6	15.985^***^	0.411^***^	0.761^***^
Escape3	0.511	0.500	51.1	24.511^***^	0.506^***^	0.793^***^
Problem1	0.495	0.500	49.5	20.319^***^	0.464^***^	0.772^***^
Problem2	0.432	0.496	43.2	20.626^***^	0.478^***^	0.769^***^
Problem3	0.062	0.241	6.2	9.337^***^	0.437^***^	0.459^***^
Deception1	0.153	0.360	15.3	13.852^***^	0.466^***^	0.633^***^
Deception2	0.270	0.444	27.0	10.264^***^	0.307^***^	0.744^***^
Deception3	0.126	0.332	12.6	12.444^***^	0.449^***^	0.705^***^
Displacement1	0.256	0.436	25.6	19.211^***^	0.506^***^	0.753^***^
Displacement2	0.128	0.334	12.8	12.629^***^	0.449^***^	0.717^***^
Displacement3	0.243	0.429	24.3	18.005^***^	0.481^***^	0.799^***^
Conflict1	0.117	0.322	11.7	12.667^***^	0.457^***^	0.795^***^
Conflict2	0.068	0.251	6.8	9.670^***^	0.393^***^	0.732^***^
Conflict3	0.078	0.268	7.8	9.918^***^	0.374^***^	0.734^***^

*** *p < 0.001. % yes refers to the percentages of affirmative answers. r_1_ refers to the correlations between items and total scores; r_2_ refers to the correlations between items and dimensional scores*.

### The Dimensional Structure and Convergent Validity of the SMD-27 Scale

The factor structure was tested in two independent samples, sample 1 (*N* = 1,298) and sample 2 (*N* = 223). The dimensional structure of the SMD-27 scale (3 items per criterion) was tested using a second-order factor model. As shown in Table 2, this resulted in an acceptable model fit in the first sample, χ^2^(835.391)/*df* (315) = 2.709, *CFI* = 0.956, *TLI* = 0.951, *RMSEA* = 0.036 (90% *CI*: 0.033–0.039). Similarly, in the second sample, the same model also showed an acceptable model fit, χ^2^(429.996)/*df* (315) =1.365, *CFI* = 0.970, *TLI* = 0.967, *RMSEA* = 0.040 (90% *CI*: 0.030–0.050). Table 3 showed the factor loadings of all 27 items in samples 1 and 2, and almost all items with factor loading exceeded 0.5 except for deception 2. Overall, the model fit and factor loadings confirmed a solid structural validity.

**Table 2:** Confirmatory factor analysis of the SMD-27 scale.

	*****χ^2^*****	***df***	*****χ^2^**/df***	***CFI***	***TLI***	***RMSEA* (90% *CI*)**
Model 1 (*N* = 1,298)	835.391	315	2.709	0.956	0.951	0.036 [0.033,0.039]
Model 2 (*N* = 223)	429.996	315	1.365	0.970	0.967	0.040 [0.030,0.050]

*Model 1 refers to sample 1; Model 2 refers to sample 2*.

*CFI, comparative fit index; TLI, Tucker-Lewis index; RMSEA, root mean square error of approximation*.

**Table 3:** The factor loadings of the SMD-27 scale.

**Item**	**Sample1** ** (*N* = 1,298)**	**Sample2** ** (*N* = 223)**	**Item**	**Sample1** ** (*N* = 1,298)**	**Sample2** ** (*N* = 223)**
Preoccupation1	0.579	0.647	Problem1	0.576	0.559
Preoccupation2	0.705	0.750	Problem2	0.597	0.638
Preoccupation3	0.667	0.718	Problem3	0.839	0.878
Tolerance1	0.735	0.826	Deception1	0.775	0.868
Tolerance2	0.700	0.711	Deception2	0.421	0.491
Tolerance3	0.801	0.865	Deception3	0.770	0.841
Withdrawal1	0.772	0.857	Displacement1	0.784	0.733
Withdrawal2	0.908	0.969	Displacement2	0.815	0.930
Withdrawal3	0.813	0.833	Displacement3	0.765	0.938
Persistence1	0.828	0.928	Conflict1	0.834	0.897
Persistence2	0.862	0.927	Conflict2	0.839	0.875
Persistence3	0.864	0.907	Conflict3	0.760	0.960
Escape1	0.874	0.855			
Escape2	0.641	0.823			
Escape3	0.786	0.867			

In terms of convergent validity, PMSMU scores showed positive correlations with the total score of the SMD-27 scale (*r* = 0.55, *p* < 0.001) and the nine dimensions' scores (*r* = 0.17–0.43, *p* < 0.001), respectively, in sample 1, indicating satisfactory convergent validity.

### Reliability of the SMD-27 Scale

The SMD-27 scale showed good internal consistency with a Cronbach's alpha of 0.87 in Sample 1 (*N* = 1,298) and 0.92 in Sample 2 (*N* = 223). The Cronbach's alpha of the nine dimensions were 0.470, 0.592, 0.669, 0.741, 0.664, 0.423, 0.457, 0.621, 0.617, respectively, in sample 1, and 0.551, 0.658, 0.773, 0.821, 0.746, 0.502, 0.589, 0.729, 0.784 in sample 2. Test-retest reliability of the SMD-27 scale was assessed among 169 students who took part in both the cross-sectional and the follow-up studies over a 1-month period in Sample 2. The Pearson correlation between the SMD-27 total score from cross-sectional study and the SMD-27 total score from follow-up study reached statistical significance (*r* = 0.60, *p* < 0.001), suggesting good test-retest reliability of the SMD-27 scale in the sample of Chinese college student.

### Impact of Social Media Disorder on Mental Health

As shown in Table 4, all correlations were significant at least at *p* < 0.001 in the expected directions. Specifically, the SMD-27 total and dimensional scores showed weak and moderate positive correlations with depression (PHQ-9) (*r* = 0.167–0.280), anxiety (IAS) (*r* = 0.207–0.303), and fatigue (FSS) (*r* = 0.078–0.267) in sample 1. The correlations between the SMD-27 scale and these related constructs indicated satisfactory criterion validity, as well as the relationship between the social media disorder and mental health.

**Table 4:** Correlations between the SMD-27 and other related scales (*N* = 1,298).

	**PHQ-9**	**IAS**	**FSS**
Preoccupation	0.280^***^	0.241^***^	0.155^***^
Tolerance	0.176^***^	0.245^***^	0.178^***^
Withdrawal	0.260^***^	0.207^***^	0.188^***^
Persistence	0.135^***^	0.240^***^	0.150^***^
Escape	0.167^***^	0.259^***^	0.260^***^
Problem	0.196^***^	0.242^***^	0.217^***^
Deception	0.226^***^	0.232^***^	0.095^***^
Displacement	0.265^***^	0.303^***^	0.169^***^
Conflict	0.206^***^	0.207^***^	0.078^***^
SMD-27	0.325^***^	0.378^***^	0.267^***^

*** *p < 0.001*.

*PHQ-9, Patient Health questionnaire-9; IAS, Interaction Anxiousness Scale; FSS, Fatigue Severity Scale*.

Findings from stepwise multiple regression analysis in sample 1 indicated that preoccupation (*B* = 0.921), displacement (*B* = 0.739), conflict (*B* = 0.914), withdrawal (*B* = 0.631) and deception (*B* = 0.463) in SMD significantly predicted depression (*F* = 51.482, *p* < 0.001), accounting for 21.8% of the variance (adjusted *R*^2^ = 0.214). Displacement (*B* = 1.620), escape (*B* = 0.840), preoccupation (*B* = 0.775), deception (*B* = 0.911), persistence (*B* = 0.556), and problems (*B* = 0.615) in SMD significantly predicted the anxiety (*F* = 37.865, *p* < 0.001), accounting for 15.0% of the variance (adjusted *R*^2^= 0.146). Escape (*B* = 1.656), problems (*B* = 1.516), and withdrawal (*B* = 1.033) in SMD significantly predicted fatigue (*F* = 29.922, *p* < 0.001), accounting for 10.4% of the variance (adjusted *R*^2^= 0.100) (Table 5).

**Table 5:** Multiple regression analysis of SMD-27 and other related scales (*N* = 1,298).

**Independent variable**	**Dependent variable**	**Unstandardized** ** coefficients**		**Standardized** ** coefficients**	***t***	***R square***	***Adjusted R square***	***F***
		***B***	***SE***	**β**				
Age	PHQ-9	1.288	0.126	0.258	10.220^***^	0.218	0.214	51.482^***^
Sex		1.384	0.357	0.099	3.879^***^			
Preoccupation		0.921	0.189	0.137	4.863^***^			
Displacement		0.739	0.197	0.109	3.760^***^			
Conflict		0.914	0.286	0.094	3.197^**^			
Withdrawal		0.631	0.186	0.096	3.402^**^			
Deception		0.463	0.217	0.059	2.137^*^			
Displacement	IAS	1.620	0.286	0.167	5.668^***^	0.150	0.146	37.865^***^
Escape		0.840	0.224	0.109	3.752^***^			
Preoccupation		0.775	0.282	0.081	2.742^**^			
Deception		0.911	0.319	0.082	2.857^**^			
Persistence		0.556	0.226	0.073	2.466^*^			
Problems		0.615	0.304	0.061	2.025^*^			
Sex	FSS	1.894	0.631	0.081	3.001^**^	0.104	0.100	29.922^***^
Age		−0.592	0.226	−0.070	−2.621^**^			
Escape		1.656	0.259	0.183	6.386^***^			
Problems		1.516	0.340	0.128	4.455^***^			
Withdrawal		1.033	0.316	0.093	3.269^**^			

* *p < 0.05*,

** *p < 0.01*,

*** *p < 0.001*.

*PHQ-9, Patient Health questionnaire-9; IAS, Interaction Anxiousness Scale; FSS, Fatigue Severity Scale*.

## Discussion

This study has validated the Chinese version of the 27-item SMD scale in Chinese college students, and it demonstrated good psychometric properties. Item analysis showed that each item had a significant positive correlation with the total score of the scale. There were significant differences between the low and the high group of college students in all items of the SMD-27 scale, indicating that the scale had good homogeneity. Moreover, the Cronbach's alpha and item factor loadings also supported the good internal consistency reliability of the scale. In addition, the SMD-27 scale also showed good test-retest reliability.

Regarding validity, the structural validity was firstly assessed based on two independent samples. The results confirmed the original second-order model of the instrument, since the indices obtained from confirmatory factor analysis showed an excellent fit of the model to the two datasets. Convergent validity was determined by the strength of correlations between SMD-27 and similar constructs. As expected, the SMD-27 scale showed strong correlations with the PMSMU.

Correlation analysis showed that both the total score and dimensional scores of the SMD-27 scale were positively correlated with depression, anxiety and fatigue. These findings demonstrated the scale's good criterion validity and further verified the close relationship between SMD and mental health (29, 30). It was suggested that when individuals regarded social media as an important or even the only way to alleviate negative emotions or compensate for attachment needs, the use of social media would gradually transition from normal to problematic mode, which would reduce the individuals' psychological defense and eventually lead to mental illness. Therefore, it is necessary for college students to have a correct understanding of social media and to learn to use social media reasonably.

There are several limitations in the study. First, the current study recruited only college students; therefore, our findings cannot be applied to those in other life stages (e.g., the elderly). Second, we used self-reported scales and the answers might be biased by social desirability. Third, we included only Chinese participants, which makes it difficult for us to compare the SMD between Western and Eastern countries directly or to examine the measurement invariance across different cultures.

With the worldwide prevalence of Internet-related addictions among adolescents, it is essential to provide a reliable and proven tool for the assessment and cross-cultural validation of this phenomenon. The present study showed that the SMD-27 scale is applicable to Chinese college populations. This scale possesses good psychometric properties in discrimination, criterion validity and construct validity in the assessment of social media addiction. Future studies are needed to verify the validity of the Chinese version of SMD-27 scale in the assessment of social media addictive behaviors in a broader population in China.

## Data Availability Statement

The raw data supporting the conclusions of this article will be made available by the authors, without undue reservation.

## Ethics Statement

The studies involving human participants were reviewed and approved by the Academic Committee of the College of Education of Hunan Agricultural University. The patients/participants provided their written informed consent to participate in this study.

## Author Contributions

HL and YH drafted the article and analyzed data. YC revised the draft. XZ designed research. All authors contributed to data interpretation and approved the final version of the manuscript.

## Funding

This study was supported by grants from the National Natural Science Foundation of China, China (Grant Number: 31600893).

## Conflict of Interest

The authors declare that the research was conducted in the absence of any commercial or financial relationships that could be construed as a potential conflict of interest.

## Publisher's Note

All claims expressed in this article are solely those of the authors and do not necessarily represent those of their affiliated organizations, or those of the publisher, the editors and the reviewers. Any product that may be evaluated in this article, or claim that may be made by its manufacturer, is not guaranteed or endorsed by the publisher.
